# The novel polymyxin analogue SPR206 exhibits higher activity than colistin against both colistin-susceptible and colistin-resistant strains of *Acinetobacter baumannii*

**DOI:** 10.1128/aac.01940-24

**Published:** 2025-05-20

**Authors:** Michelle Outeda-García, Andrea Garcia-Pose, Paula Guijarro-Sánchez, Arianna Rodríguez-Coello, Gabriela Alejandra Báez-Barroso, Romina Maceiras, Isaac Alonso-García, Jorge Arca-Suárez, Juan C. Vázquez-Ucha, German Bou, Alejandro Beceiro, on behalf of the Spanish National Acinetobacter spp. 2020 Study Group

**Affiliations:** 1 Microbiology Service and Institute for Biomedical Research A Coruña (INIBIC), Complexo Hospitalario Universitario A Coruña (CHUAC) 16811, A Coruña, Spain; 2 CIBER de Enfermedades Infecciosas (CIBERINFEC), Instituto Salud Carlos III 637284 , Madrid, Spain; 3 Department of Physiotherapy, Medicine and Biomedical Sciences, University of A Coruña 16737, A Coruña, Spain; University of Fribourg, Fribourg, Switzerland

**Keywords:** antimicrobial resistance, colistin, SPR206, *Acinetobacter baumannii*

## Abstract

Colistin resistance is increasing globally and complicates treatments of *A. baumannii* infections. The next-generation polymyxin SPR206 shows potent activity against multidrug-resistant Gram-negative pathogens with low toxicity. SPR206 exhibited higher activity against colistin-susceptible and colistin-resistant strains (MIC_50_/MIC_90_ = 0.12/0.25 mg/L), and resistance was only detected in 1/118 strains (MIC ≥4 mg/L). Mutations in *pmrCAB*, linked to colistin resistance, do not seem to confer resistance to the novel polymyxin, which retains a high level of activity.

## INTRODUCTION

*Acinetobacter baumannii* is a nosocomial pathogen associated with a wide range of infections, particularly ventilator-associated pneumonia and bacteremia. Carbapenem-resistant *A. baumannii* is renowned for its ability to acquire antimicrobial resistance (1), and it was therefore classified by the World Health Organization (WHO) as a critical priority pathogen for new drug development in 2024 (2). Colistin (polymyxin E) is considered a “last-resort” drug for treating *A. baumannii* infections; however, despite its potent antimicrobial activity, its use is associated with significant drawbacks, including high nephrotoxicity, neurotoxicity, and challenging PK/PD properties (3). Furthermore, the increasing incidence of colistin resistance in recent years highlights the urgent need for new antimicrobials to combat *A. baumannii* infections (4).

**Fig 1:**
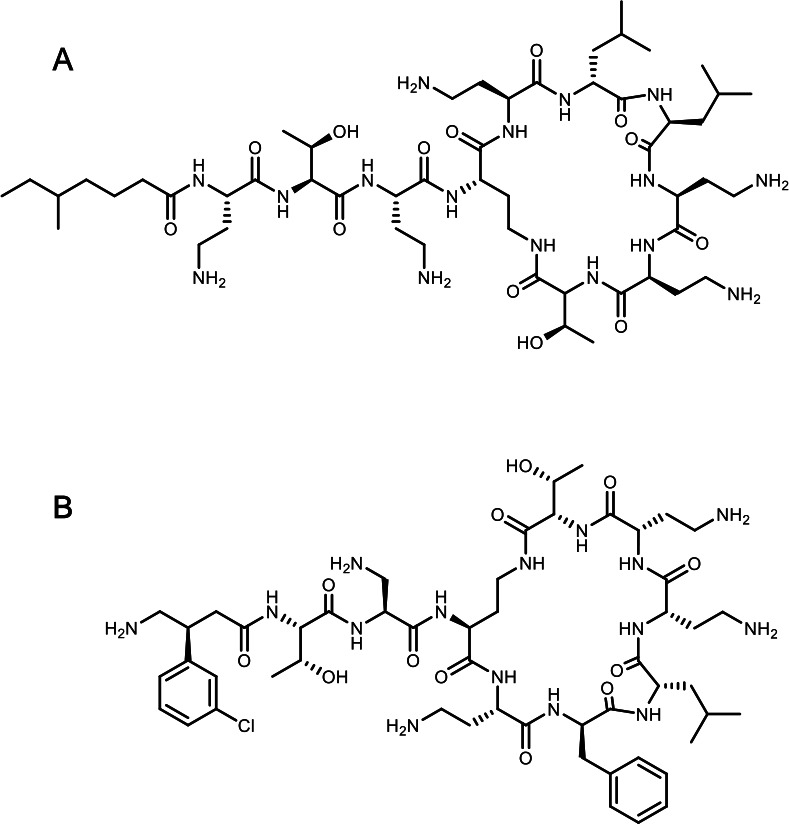
Chemical structures of colistin (A) and SPR206 (B).

In response to this situation, several new polymyxin analogs with promising activity have been developed. Among these, SPR206 (also known as EVER206) exhibits potent activity against *Enterobacterales*, *Pseudomonas aeruginosa*, and *A. baumannii*, as well as significantly lower toxicity (5–7). SPR206, which shows structural similarities to colistin, has a diaminopropionate residue adjacent to the cyclic core (Fig. 1) and is designed to maintain potent polymyxin activity by destabilizing the bacterial outer membrane, ultimately resulting in cell lysis. The optimized structure enhances the compound’s selectivity for the bacterial membrane, reducing interactions with human cell membranes (5). The safety and pharmacokinetics of this next-generation polymyxin in humans have been evaluated in several Phase 1 clinical trials, with promising results. FDA approval has recently been given for Phase 2 trials to be undertaken to evaluate the efficacy of the compound against carbapenem-resistant *A. baumannii* and *P. aeruginosa* infections (8). Although SPR206 has demonstrated high activity against *A. baumannii*, including carbapenem-resistant strains (7), its activity against colistin-resistant strains has scarcely been studied. The activity of SPR206 is not well understood, and the extent to which it is affected by resistance mechanisms that limit other classical polymyxins, such as colistin, is not clear. Further evaluation of the activity of SPR206 by using large, fully sequenced multicenter collections is essential to comprehensively define its therapeutic range and limitations. Such studies will also provide valuable insights to inform strategies aimed at mitigating the emergence and spread of antimicrobial resistance and guiding the clinical use of the compound for difficult-to-treat pathogens.

In this study, the activity of SPR206 and colistin was tested against *A. baumannii* strains with different and well-characterized colistin resistance mechanisms, including lipid A modifications (e.g., *pmrB*) and the less frequent deficits in LPS biosynthesis. Three isogenic pairs of colistin-susceptible and colistin-resistant clinical isolates (ABRIM/ABRIM*pmrB*, GR.AB248/GR.AB249*pmrB*, and GR.AB299/GR.AB347*pmrB*) were included, with the last two pairs collected consecutively from two intensive care unit patients, pre- and post- colistin treatment (9). The *A. baumannii* type strain ATCC 19606 was also included, along with its isogenic colistin-resistant mutants: ATCC 19606*pmrB*, ATCC 19606*ΔlpxA*, ATCC 19606Δ*lpxC*, and ATCC 19606Δ*lpxD* (10). The molecular mechanisms of colistin resistance of these strains have been extensively characterized in previous studies (Table 1). A total of 118 sequenced clinical strains of *A. baumannii*, isolated in a Spanish national multicenter study, conducted in 2020 and involving more than 20 hospitals (4), were also included. In addition, 12 colistin-resistant *A. baumannii* isolates, 4 from the Institute for Global Health (Barcelona, Spain), and 8 from the previous Spanish National *A. baumannii* 2010 Study were tested against both polymyxins.

**TABLE 1:** Description of *A. baumannii* strains and MICs of colistin and SPR206 (mg/L)

*A. baumannii* strain	Colistin	SPR206	Description	Source or reference
ATCC 17978	0.5	0.03	*A. baumannii* reference strain	ATCC
ABRIM	0.5	0.06	*A. baumannii* clinical strain	(11)
ABRIM*pmrB*	32	1	Isogenic derivative mutant of ABRIM; single amino acid substitution (Asn353Tyr) in PmrB	(12)
GR.AB299	0.5	0.03	*A. baumannii* clinical strain	(9)
GR.AB347*pmrB*	64	2	Isogenic clinical strain derivative of GR.AB299; single amino acid substitution (Pro170Leu) in PmrB	(9)
GR.AB248	0.5	0.03	*A. baumannii* clinical strain	(9)
GR.AB249*pmrB*	256	4	Isogenic clinical strain derivative of GR.AB248; single amino acid substitution (Pro233Ser) in PmrB	(9)
ATCC 19606	0.5	0.06	*A. baumannii* type strain	ATCC
ATCC 19606*pmrB*	32	2	Isogenic derivative mutant of ATCC 19606; single amino acid substitution (Ala227Val) in PmrB	(12)
ATCC 19606Δ*lpxA*	256	≥256	Isogenic derivative mutant of ATCC 19606; 445 bp deletion at nucleotide 364 within the *lpxA* gene and frameshift after His121	(13)
ATCC 19606Δ*lpxC*	1024	≥256	Isogenic derivative mutant of ATCC 19606; 84 bp deletion at nucleotide 858 within the *lpxC* gene and frameshift after Thr285	(13)
ATCC 19606Δ*lpxD*	1024	≥256	Isogenic derivative mutant of ATCC 19606; single-base deletion at nucleotide 952 of the *lpxD* gene and frameshift after Lys317	(13)

The minimum inhibitory concentrations (MICs) and minimum bactericidal concentrations (MBCs) of colistin and SPR206 were determined using broth microdilution, following CLSI criteria (14), and MIC_50_/MIC_90_ values and resistance rates were calculated. Time–kill curve analyses were conducted using colistin and SPR206 at concentrations of 0.5×, 1×, and 2× MIC with two pairs of isogenic clinical isolates: GR.AB299/GR.AB347*pmrB* and GR.AB248/GR.AB249*pmrB. A. baumannii* cultures were grown overnight, and 3 × 10⁵ CFUs/mL were used to inoculate 150 µL of Mueller-Hinton II broth in 96-well plates. Growth was monitored for 48 h using an Epoch 2 Microplate Spectrophotometer (BioTek), and bacterial counts (CFU/mL) were determined after 0, 2, 4, 8, 24, and 48 h, by plating the strains onto Mueller-Hinton agar plates.

Comparative WGS analysis was performed for colistin-resistant and phylogenetically related susceptible clinical isolates using Panaroo (15) for presence/absence analysis, Snippy (16) for SNP detection, and manual curation for IS*Aba* elements identification. Previously described resistance mechanisms and membrane-related elements were systematically analyzed as detailed in Table S1. Core genome multilocus sequence typing (cgMLST) was performed using ChewBBACA with the public *A. baumannii* schema at a 95% presence threshold. The allelic profiles were used to construct a Neighbor-Joining tree with GrapeTree based on allelic differences. The resulting Newick tree was visualized using the *ggtree* package in R.

SPR206 exhibited higher *in vitro* activity than colistin against the full panel of colistin-susceptible strains, with lower MICs (8- to 16-fold lower). Notably, SPR206 also demonstrated significantly higher activity against colistin-resistant strains due to *pmrB* mutations, yielding lower MICs (16- to 32-fold lower). However, SPR206 did not improve colistin activity against the strains resistant to this antibiotic due to alterations in the *lpx* genes (MICs ≥256 mg/L for both antibiotics against all strains), likely reflecting the absence of lipid A in these mutants, which is the target structure of polymyxins (Table 1).

The MIC_50_/MIC_90_ values were significantly lower for the novel polymyxin than for colistin (0.12/0.25 mg/L and 1/4 mg/L, respectively) against the entire set of 118 isolates (Table 2). Applying the colistin resistance breakpoints recommended by CLSI and EUCAST (MIC ≥4 mg/L) to both antibiotics, the *A. baumannii* collection exhibited 11.9% resistance to colistin (14 strains with MICs ≥ 4 mg/L), but only 0.8% resistance to SPR206 (1 strain, MIC = 8 mg/L). The phylogenetic tree based on cgMLST analysis of the 14 colistin-resistant strains showed that they belonged to diverse sequence types and genetic lineages, supporting their epidemiological unrelatedness (Fig. S1).

**TABLE 2:** Cumulative distribution of the minimum inhibitory concentration (MIC) and percentage of resistance (MIC ≥4 mg/L) to colistin and SPR206 relative to the set of *A. baumannii* strains isolated in a national multicenter study^*b*^

Antimicrobial	Cumulative % of strains at MIC in mg/L (num. of total strains)^*c*^												% of resistance
	≤0.03	0.06	0.12	0.25	0.5	1	2	4	8	16	32	≥ 64	
All sets of strains (*N* = 118)													
Colistin			0.9 (1)^*a*^	5.9 (7)	33.9 (40)	83.9 (99)	88.1 (104)	**94.1** (111)	99.2 (117)	99.2 (117)	99.2 (117)	100 (118)	11.9
SPR206	9.3 (11)^*a*^	32.2 (38)	72.8 (86)	**97.5** (115)	99.2 (117)	99.2 (117)	99.2 (117)	99.2 (117)	100 (118)				0.8
Colistin-susceptible strains (*N* = 104)													
Colistin			1.0 (1)	6.7 (7)	38.5 (40)	**95.2** (99)	100 (104)						0
SPR206	9.6 (10)^*a*^	34.6 (36)	76.0 (79)	**98.1** (102)	100 (104)								0
Colistin-resistant strains (*N* = 14)													
Colistin								50.0 (7)	**92.9** (13)	92.9 (13)	92.9 (13)	100 (14)	100
SPR206	7.1 (1)^*a*^	14.3 (2)	50 (7)	**92.9** (13)	92.9 (13)	92.9 (13)	92.9 (13)	92.9 (13)	100 (14)				7.1

^*a*^ Actual MIC value is equal to or inferior to that indicated in the table.

^*b*^ MIC_50_ underlined, MIC_90_ in bold.

^*c*^ Empty cells indicate that the MIC was not evaluated at that specific concentration for that group of strains.

The robust activity of SPR206 against colistin-susceptible isolates of *A. baumannii* has previously been described, with SPR206 and colistin MIC_50_/MIC_90_ values of 0.06/0.12 mg/L and 0.25/0.25 mg/L, respectively, in a small collection of 20 isolates (7). Focusing on the comparative analysis of the activity of SPR206 and colistin against the colistin-susceptible strains here (104/118) (Table 2), we obtained MIC_50_/MIC_90_ values of 0.12/0.25 mg/L and 1/1 mg/L for SPR206 and colistin, respectively. Therefore, both studies revealed that SPR206 was more active than colistin against colistin-susceptible strains, with MIC_50_/MIC_90_ values 4- to 8-fold lower. Higher activity of SPR206 was also observed in another recent study using a collection of 30 clinical strains of *A. baumannii*, including 17 colistin-resistant strains (17). Considering only the 17 colistin-resistant strains included in that study, MIC_50_/MIC_90_ values of 1/16 mg/L for SPR206 and 16/256 mg/L for colistin were obtained. In the present study, analysis of the 14 colistin-resistant isolates yielded MIC_50_/MIC_90_ values of 0.12/0.25 mg/L for SPR206 and 4/8 mg/L for colistin (Table 2). Thus, both studies demonstrated the excellent activity of SPR206 against colistin-resistant strains (MIC values 16- to 32-fold lower). The differences in the MIC values obtained in both studies are probably due to the genetic diversity and the underlying mechanisms of colistin resistance of the strains included in the collections. Interestingly, the isolate with the highest resistance to colistin in our study (MIC ≥64 mg/L) was also the isolate with the highest SPR206 MIC (8 mg/L).

In order to confirm the potent activity of SPR206 against colistin-resistant *A. baumannii* strains, the MIC of 12 additional isolates from two other different collections was also evaluated. This pool of 12 colistin-resistant *A. baumannii* strains showed MIC₅₀/MIC₉₀ values for colistin of 16/32 mg/L and for SPR206 of 0.12/1 mg/L (Table S3), concordant with the MICs obtained for the strains above. Thus, when combined with the 14 strains from the Spanish National 2020 Study collection (total 26 strains), the resulting MIC₅₀/MIC₉₀ values were 8/32 mg/L for colistin and 0.12/1 mg/L for SPR206, confirming the higher potency of SPR206 against resistant isolates (Table S4).

Later, the MBC, defined as the lowest drug concentration that reduces the initial bacterial inoculum by ≥99.9% (18), was assessed for colistin-resistant *A. baumannii* isolates carrying *pmrAB*-dependent resistance mechanisms, as well as their colistin-susceptible parental strains. MBC determinations matched MIC values for all strains (displayed in Table 1), confirming a potent bactericidal activity of SPR206, similar to that previously described for colistin (19).

In order to evaluate the stability of this new polymyxin, time–kill assays were performed using two isogenic clinical pairs of colistin-susceptible and colistin-resistant *A. baumannii*. As shown in Fig. S2, SPR206 exhibits sustained antimicrobial activity over a 48-hour period when used at concentrations close to the MIC (0.5×, 1 ×, and 2 × MIC), similar to that shown by colistin. Of note, however, SPR206 was administered at absolute concentrations significantly lower than those used for colistin (16- to 64-fold lower).

WGS analysis of the colistin resistance mechanisms in the 14 colistin-resistant strains included in the 2020 collection revealed that 4 strains had mutations in the *pmrABC* genes (Table S2). Mutations in *pmrABC* genes have previously been associated with the addition of phosphoethanolamine residues to lipid A, which reduces the LPS charge and increases the resistance to colistin (12, 20). Analysis of the isolate that yielded the highest colistin and SPR206 MICs (isolate AB171) revealed a nonfunctional Tim44 lipid-binding transport protein (frameshift Lys146fs), along with a ~10 kb deletion encompassing transcriptional regulators (*irp* and *acrR*), efflux transporters (*rhtA* and *araJ*), and an acyltransferase (annotated as lauroyl acyltransferase/LpxL by homology with *Pseudomonas* sp.). Given the size of the deletion and the diverse functions of the affected genes, multiple factors in this region may be contributing to the observed high-level colistin resistance (MIC ≥ 64 mg/L). Notably, even this highly resistant isolate with significant membrane-related alterations had moderately elevated SPR206 MIC to 8 mg/L, thus highlighting the increased stability of SPR206 against these colistin resistance mechanisms.

The underlying mechanism of resistance in the remaining nine colistin-resistant strains remains unclear. Other genes, such as *mcr* (PEtN transferase), *basRS* (two-component signal transduction system), outer membrane proteins, and others involved in membrane modifications, have been implicated in colistin resistance in Gram-negative pathogens (20, 21) (Table S1); however, no *mcr* genes or changes in the coding sequences of these proteins or in the promoter regions were identified.

These results, together with those obtained with the isogenic colistin-resistant mutants, suggest that SPR206 retains substantial activity against strains harboring *pmrB* mutations, despite the associated high-level colistin resistance. However, further detailed studies are required to elucidate the underlying causes of reduced susceptibility to SPR206 in *A. baumannii*. Similarly, we observed, for the first time, that the complete loss of LPS caused by mutations in the *lpx* genes confers high resistance to both colistin and SPR206. The total loss of LPS due to alterations in *lpx* genes is rarely identified in clinical isolates, probably due to the high fitness cost, thus suggesting that the emergence of *lpx*-mediated resistance is expected to scarcely occur (22).

In conclusion, our study findings demonstrate the improved activity of SPR206 against both colistin-susceptible and colistin-resistant strains of *A. baumannii*. In addition, high activity against both clinical strains and isogenic strains with specific mutations in *pmrCAB* that confer colistin resistance was observed. However, mutations in *lpx* genes, which are infrequent among colistin-resistant strains of clinical origin, compromised the activity of SPR206. Due to its limited toxicity and its ability to overcome colistin resistance, SPR206 could potentially be considered a therapeutic option for treating infections caused by *A. baumannii*, particularly in cases where current treatments are limited due to resistance or toxicity.

## Data Availability

The data supporting this work are available under NCBI bioproject PRJNA991768.

## SUPPLEMENTAL MATERIAL

Supplemental material aac.01940-24-s0001.docx Tables S1 to S4; Fig. S1 and S2.

ASM does not own the copyrights to Supplemental Material that may be linked to, or accessed through, an article. The authors have granted ASM a non-exclusive, world-wide license to publish the Supplemental Material files. Please contact the corresponding author directly for reuse.
