# Supplementary material for: The novel polymyxin analogue SPR206 exhibits higher activity than colistin against both colistin-susceptible and colistin-resistant strains of Acinetobacter baumannii
Source: Antimicrob Agents Chemother. 2025 May 20;69(7):e01940-24. doi: 10.1128/aac.01940-24 (PMC12217451; doi:10.1128/aac.01940-24)

**Title:**

**The novel polymyxin analogue SPR206 exhibits higher activity than colistin against both colistin-susceptible and colistin-resistant strains of *Acinetobacter baumannii*.**

**Authors:**

Michelle Outeda-García^a,¥^, Andrea Garcia-Pose^a,¥^, Paula Guijarro-Sánchez^a^, Arianna Rodríguez-Coello^a^, Gabriela Alejandra Báez-Barroso^a^, Romina Maceiras^a^, Isaac Alonso-García^a^, Jorge Arca-Suarez^a,b^, Juan C. Vázquez-Ucha^a,b,^*, German Bou^a,b,c†^ and Alejandro Beceiro^a,b,†^ on behalf of the Spanish National *Acinetobacter* spp. 2020 Study Group ^‡^.

^a^ Servicio de Microbiología and Instituto de Investigación Biomédica A Coruña (INIBIC), Complexo Hospitalario Universitario A Coruña (CHUAC), A Coruña, Spain

^b^ Ciber de Enfermedades Infecciosas (CIBERINFEC), Instituto de Salud Carlos III, Madrid, Spain

^c^ Departamento de Fisioterapia, Medicina y Ciencias Biomédicas, Universidad de A Coruña, Spain

**Suplementary Table 1**_________________________Page 2

**Suplementary Table 2**_________________________Page 3

**Suplementary Table 3**_________________________Page 6

**Suplementary Table 4**_________________________Page 6

**Suplementary Figure 1**________________________Page 7

**Suplementary Figure 2**________________________Page 8

**Suplementary Table 1.** Detailed analytical strategy used to determine colistin resistance mechanisms. Gene disruption analysis includes identification of premature stop codons, frameshift mutations from variant calling, and gene interruption by ISAba elements. Upstream regions analysis includes searching for ISAba elements that may affect gene expression.

| Mechanism | Genes/Elements | SNP analysis | Presence/Absence | *ISAba* analysis | Other modifications |
| --- | --- | --- | --- | --- | --- |
| Loss of lipid A biosynthesis | Lpx genes | Yes | Yes | Upstream regions | Gene disruption |
|  | Other lipid biosynthesis -related mechanisms | Yes | Yes | Upstream regions | Gene disruption |
| Phosphoethanolamine addition to LPS | pmrABC | Yes | Yes | Upstream regions | - |
| Plasmid-mediated resistance (phosphoethanolamine transferase) | mcr family | No | Yes | No | - |
| Other membrane modifications | Lipoproteins | No | Yes | Upstream regions | Gene disruption |
|  | Transport proteins | No | Yes | Upstream regions | Gene disruption |
|  | Membrane integrity proteins | No | Yes | Upstream regions | Gene disruption |
| Regulatory elements | Transcriptional regulators | Yes | Yes | Upstream regions | Gene disruption |
|  | Two-component systems | Yes | Yes | Upstream regions | - |
| Outer membrane porins (OMPs) alterations | OmpA, CarO, OprD-like, OmpW, Omp33-36 | Yes | Yes | No | Gene disruption |

**Supplementary Table 2.** Isolate number, city of origin, sequence type (ST), MIC values (mg/L) and potential mechanism of colistin resistance for *A. baumannii* strains (n=118).

| Strain and  genome number | City | ST | Colistin | SPR206 | Potential mechanism of colistin resistance |
| --- | --- | --- | --- | --- | --- |
| AB1 | A Coruña | 164 | 1 | 0.25 |  |
| AB4 | Sevilla | 2 | 1 | 0.12 |  |
| AB5 | Sevilla | 2 | 1 | 0.12 |  |
| AB6 | Sevilla | 2 | 1 | 0.12 |  |
| AB7 | Sevila | 2 | 1 | 0.12 |  |
| AB8 | Sevilla | 2 | 1 | 0.12 |  |
| AB9 | Granada | 2 | 1 | 0.12 |  |
| AB10 | Sevilla | 2 | 0.5 | 0.12 |  |
| AB11 | A Coruña | 239 | 1 | 0.12 |  |
| AB12 | Tenerife | 2 | 1 | 0.12 |  |
| AB13 | Tenerife | 2 | 1 | 0.25 |  |
| AB14 | Tenerife | 2 | 1 | 0.12 |  |
| AB15 | Tenerife | 2 | 1 | 0.25 |  |
| AB16 | Tenerife | 2 | 1 | 0.12 |  |
| AB17 | Tenerife | 2 | 4 | 0.25 |  |
| AB18 | Tenerife | 2 | 2 | 0.12 |  |
| AB19 | Tenerife | 2 | 2 | 0.25 |  |
| AB20 | Tenerife | 2 | 1 | 0.12 |  |
| AB24 | Tenerife | 2 | 1 | 0.25 |  |
| AB25 | Tenerife | 2 | 1 | 0.12 |  |
| AB26 | Tenerife | 2 | 4 | 0.25 | PmrB Q34P |
| AB28 | Valencia | 2 | 8 | 0.12 |  |
| AB29 | Valencia | 2 | 1 | 0.25 |  |
| AB30 | Valencia | 2 | 8 | 0.12 |  |
| AB31 | Valencia | 2 | 1 | 0.12 |  |
| AB32 | Valencia | 2 | 8 | 0.12 |  |
| AB33 | Valencia | 2 | 1 | 0.25 |  |
| AB34 | Valencia | 2182 | 0.5 | 0.12 |  |
| AB35 | Valencia | 2 | 8 | 0.25 |  |
| AB36 | Valencia | 2 | 1 | 0.12 |  |
| AB37 | Valencia | 2 | 8 | 0.25 |  |
| AB38 | Valencia | 866 | 1 | 0.12 |  |
| AB39 | Valencia | 2 | 1 | 0.25 |  |
| AB40 | Valencia | 2176 | 1 | 0.25 |  |
| AB41 | Valencia | 2 | 4 | 0.25 |  |
| AB42 | Valencia | 2 | 8 | 0.25 |  |
| AB44 | Sevilla | 2 | 0.5 | 0.12 |  |
| AB45 | Sevilla | 1 | 1 | 0.12 |  |
| AB46 | Sevilla | 1 | 1 | 0.12 |  |
| AB47 | Sevilla | 1 | 1 | 0.12 |  |
| AB49 | Sevilla | 1 | 1 | 0.12 |  |
| AB50 | Sevilla | 2176 | 0.5 | 0.12 |  |
| AB52 | Sevilla | 1 | 0.5 | 0.12 |  |
| AB53 | Sevilla | 1 | 1 | 0.25 |  |
| AB54 | Sevilla | 1 | 1 | 0.12 |  |
| AB55 | Sevilla | 1 | 1 | 0.12 |  |
| AB56 | Sevilla | 1 | 1 | 0.12 |  |
| AB57 | Lleida | 1470 | 1 | 0.12 |  |
| AB58 | Lleida | 6 | 1 | 0.25 |  |
| AB61 | Lleida | 1639 | 1 | 0.12 |  |
| AB62 | Lleida | 265 | 1 | 0.25 |  |
| AB67 | Lleida | 1 | 2 | 0.25 |  |
| AB79 | Bilbao | 106 | 1 | 0.25 |  |
| AB80 | Bilbao | 2182 | 0.5 | ≤0.03 |  |
| AB81 | Bilbao | 25 | 1 | 0.5 |  |
| AB83 | Bilbao | 25 | 1 | 0.25 |  |
| AB93 | Bilbao | 25 | 1 | 0.5 |  |
| AB95 | Ferrol | 582 | 0.5 | 0.25 |  |
| AB96 | Ferrol | 582 | 0.5 | 0.25 |  |
| AB100 | Vitoria | 145 | 0.5 | 0.06 |  |
| AB109 | Córdoba | 1 | 2 | 0.06 |  |
| AB110 | Córdoba | 1623 | 4 | 0.12 | PmrC A46T |
| AB112 | Córdoba | 193 | 2 | 0.25 |  |
| AB124 | Badalona | 2176 | 1 | 0.06 |  |
| AB127 | Badalona | 2176 | 0.5 | 0.06 |  |
| AB128 | Badalona | 2176 | 1 | 0.25 |  |
| AB130 | A Coruña | 32 | 0.5 | 0.25 |  |
| AB131 | Tarragona | 687 | 1 | 0.12 |  |
| AB132 | Tarragona | 1112 | 1 | 0.06 |  |
| AB133 | Tarragona | 2 | 1 | ≤0.03 |  |
| AB134 | Tarragona | 2 | 1 | 0.12 |  |
| AB136 | A Coruña | 2183 | 1 | 0.12 |  |
| AB137 | Cádiz | 85 | 0.25 | ≤0.03 |  |
| AB138 | Cádiz | 745 | 1 | ≤0.03 |  |
| AB139 | Cádiz | 54 | 0.25 | 0.06 |  |
| AB140 | Cádiz | 85 | ≤0.12 | ≤0.03 |  |
| AB141 | Cádiz | 745 | 1 | 0.06 |  |
| AB142 | Cádiz | 1 | 0.5 | 0.06 |  |
| AB143 | Cádiz | 1328 | 1 | 0.06 |  |
| AB144 | Cádiz | 745 | 0.5 | 0.06 |  |
| AB148 | Cádiz | 745 | 1 | 0.06 |  |
| AB149 | Cádiz | 1112 | 1 | 0.06 |  |
| AB150 | Cádiz | 2184 | 0.5 | ≤0.03 |  |
| AB152 | Cádiz | 132 | 0.5 | 0.12 |  |
| AB158 | Palma de Mallorca | 2034 | 0.5 | 0.06 |  |
| AB163 | Palma de Mallorca | 132 | 0.5 | 0.06 |  |
| AB167 | Oviedo | 1623 | 0.5 | 0.06 |  |
| AB168 | Oviedo | 1470 | 0.25 | 0.12 |  |
| AB169 | Oviedo | 2 | 1 | 0.12 |  |
| AB170 | Oviedo | 2110 | 1 | 0.06 |  |
| AB171 | Oviedo | 2 | ≥64 | 8 | Loss of *lpxL*-like (lipid A biosynthesis lauroyl acyltransferase) and other membrane integrity genes |
| AB172 | Oviedo | 2185 | 0.5 | ≤0.03 |  |
| AB173 | Oviedo | 2 | 4 | 0.06 |  |
| AB174 | Oviedo | 2 | 0.5 | 0.06 |  |
| AB179 | Murcia | 2 | 0.5 | ≤0.03 |  |
| AB180 | Murcia | 2 | 4 | ≤0.03 | PmrB L94M |
| AB181 | Murcia | 2 | 0.5 | ≤0.03 |  |
| AB182 | Murcia | 2 | 0.5 | 0.06 |  |
| AB183 | Murcia | 2 | 0.5 | 0.06 |  |
| AB184 | Murcia | 2 | 0.5 | 0.06 |  |
| AB185 | Murcia | 2 | 1 | 0.12 |  |
| AB186 | Murcia | 2 | 0.5 | 0.06 |  |
| AB187 | Murcia | 105 | 0.5 | 0.12 |  |
| AB188 | Murcia | 203 | 4 | 0.12 | PmrA S119T |
| AB189 | Murcia | 1405 | 0.5 | 0.12 |  |
| AB190 | Murcia | 2186 | 0.5 | 0.12 |  |
| AB191 | Murcia | 2 | 0.25 | 0.06 |  |
| AB192 | Murcia | 2 | 0.5 | 0.06 |  |
| AB193 | Murcia | 164 | 0.5 | 0.12 |  |
| AB194 | Murcia | 2 | 1 | 0.06 |  |
| AB195 | Murcia | 1 | 1 | 0.25 |  |
| AB199 | Murcia | 2 | 1 | 0.12 |  |
| AB200 | Murcia | 1336 | 0.5 | 0.12 |  |
| AB201 | Murcia | 2 | 0.25 | 0.25 |  |
| AB202 | Murcia | 1 | 1 | 0.06 |  |
| AB203 | Murcia | 221 | 0.5 | 0.25 |  |
| AB204 | Murcia | 2 | 0.25 | ≤0.03 |  |
| AB206 | A Coruña | 2187 | 1 | 0.06 |  |

**Supplementary Table 3.** Accumulative MIC (mg/L) distributions and resistance rates to colistin and SPR206 of 12 colistin resistant *A. baumannii* strains from the i) Institute for Global Health, Barcelona, Spain (n=4), and ii) Spanish National *A. baumannii* 2010 Study (n=8).

| **Antibiotics** | **0.06** | **0.12** | **0.25** | **0.5** | **1** | **2** | **4** | **8** | **16** | **32** | **64** |
| --- | --- | --- | --- | --- | --- | --- | --- | --- | --- | --- | --- |
| **Colistin** |  |  |  |  |  |  |  | 16.6 (2) | 58.3 (6) | **91.7** (11) | 100 (12) |
| **SPR206** | 9.1 (1) | 58.3 (7) | 66.7 (8) | 75  (9) | **100** (12) |  |  |  |  |  |  |

MIC_50_ underlined, MIC_90_ in bold.

**Supplementary Table 4.** Accumulative MIC (mg/L) distributions and resistance rates to colistin and SPR206 of the total 26 colistin resistant *A. baumannii* strains used in this study: 14 from Spanish National *Acinetobacter* spp. 2020 Study, 4 from Institute for Global Health (Barcelona, Spain), and 8 from Spanish National *A. baumannii* 2010 Study.

| **Antibiotics** | **0.03** | **0.06** | **0.12** | **0.25** | **0.5** | **1** | **2** | **4** | **8** | **16** | **32** | **64** |
| --- | --- | --- | --- | --- | --- | --- | --- | --- | --- | --- | --- | --- |
| **Colistin** |  |  |  |  |  |  |  | 26.9 (7) | 57.7 (15) | 73.1 (19) | **92.3** (24) | 100 (26) |
| **SPR206** | 3.8 (1) | 11.5 (3) | 53.8 (14) | 80.8 (21) | 84.6 (22) | **96.2** (25) | 96.2 (25) | 96.2 (25) | 53.8 (14) | 100 (26) |  |  |

MIC_50_ underlined, MIC_90_ in bold.

**Supplementary Figure 1.** Phylogenetic tree of *A. baumannii* clinical isolates with antimicrobial susceptibility data. Core genome MLST-based phylogenetic tree showing the genetic relationships among 14 *A. baumannii* isolates with corresponding colistin and SPR206 MICs and resistance mechanisms.


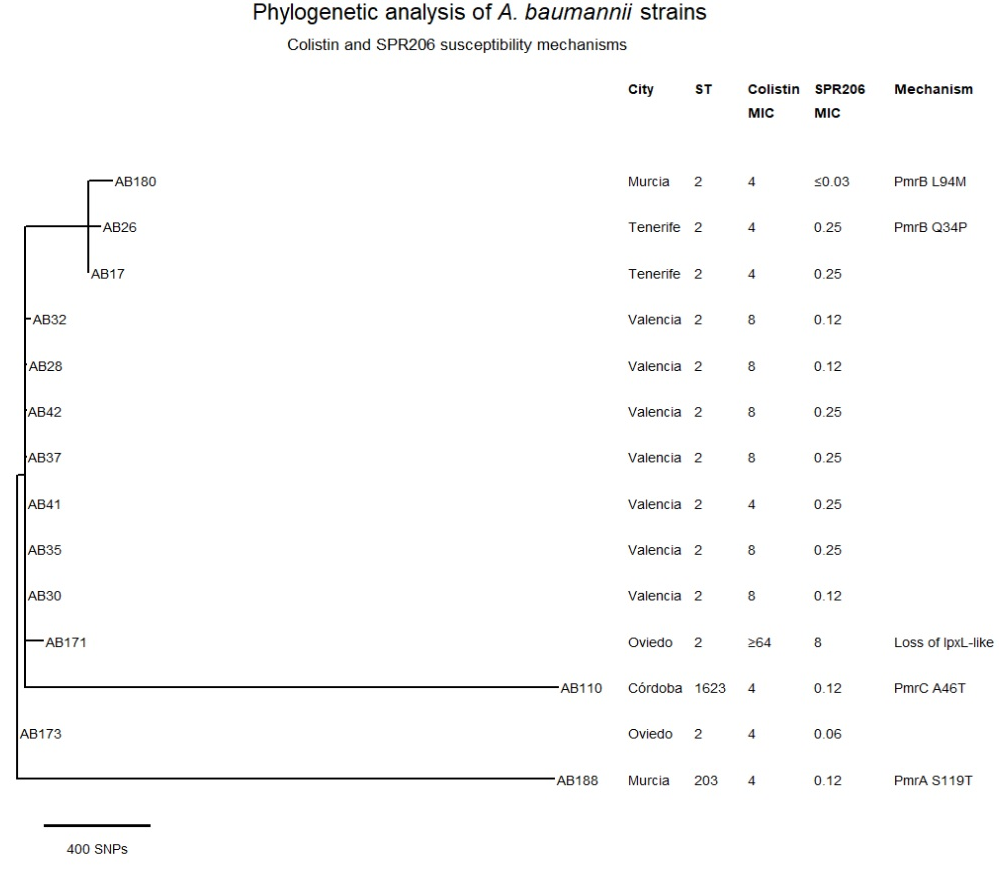


**Supplementary Figure 2**. Antimicrobial activity of colistin and SPR206 in Mueller Hinton broth. Drugs were added to 0.5x, 1x. and 2x MIC; growth control without antibiotic was also included. (a) GR.299 *A. baumannii* isolate and its isogenic clinical strain derivative GR.347*pmrB* (colistin-resistant); (b) GR.248 *A. baumannii* isolate and its isogenic clinical strain derivative GR.249*pmrB* (colistin-resistant).


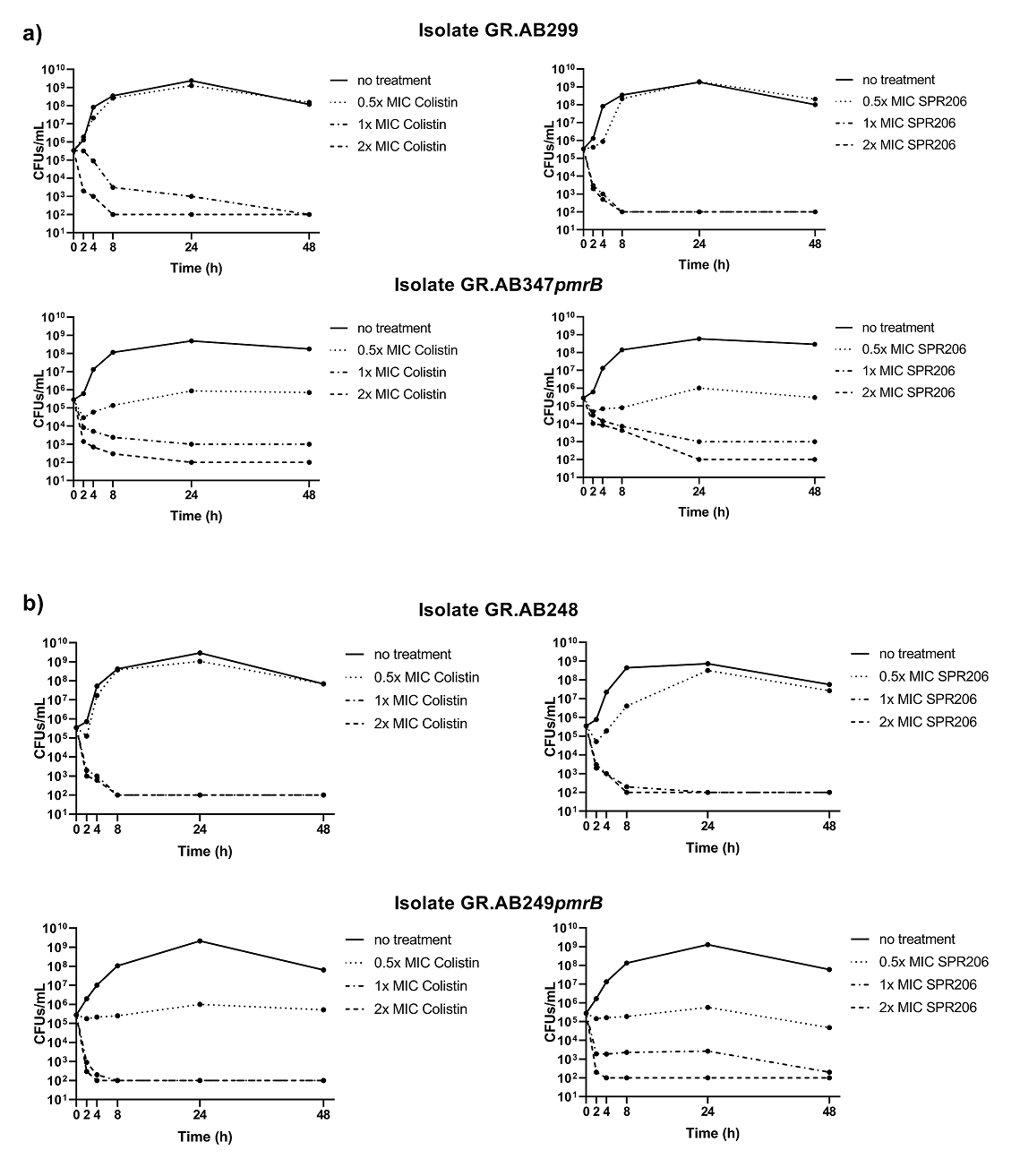

Supplement: Supplemental material — Tables S1 to S4; Fig. S1 and S2. [file aac.01940-24-s0001.docx]
